# Estimation of R_0_ for the Spread of the First ASF Epidemic in Italy from Fresh Carcasses

**DOI:** 10.3390/v14102240

**Published:** 2022-10-12

**Authors:** Federica Loi, Daria Di Sabatino, Ileana Baldi, Sandro Rolesu, Vincenzo Gervasi, Vittorio Guberti, Stefano Cappai

**Affiliations:** 1 Osservatorio Epidemiologico Veterinario Regionale della Sardegna, Istituto Zooprofilattico Sperimentale della Sardegna, 07100 Sassari, Italy; 2 Istituto Zooprofilattico Sperimentale dell’Abruzzo e del Molise G. Caporale, 64100 Teramo, Italy; 3 Department of Cardiac Thoracic Vascular Sciences and Public Health, University of Padova, 35131 Padova, Italy; 4 Institute for Environmental Protection and Research (ISPRA), 00144 Roma, Italy

**Keywords:** African swine fever, wild boar, basic reproduction number, doubling time, disease eradication, mathematical model, carcasses

## Abstract

After fifty years of spread in the European continent, the African swine fever (ASF) virus was detected for the first time in the north of Italy (Piedmont) in a wild boar carcass in December, 2021. During the first six months of the epidemic, the central role of wild boars in disease transmission was confirmed by more than 200 outbreaks, which occurred in two different areas declared as infected. The virus entered a domestic pig farm in the second temporal cluster identified in the center of the country (Lazio). Understanding ASF dynamics in wild boars is a prerequisite for preventing the spread, and for designing and applying effective surveillance and control plans. The aim of this work was to describe and evaluate the data collected during the first six months of the ASF epidemic in Italy, and to estimate the basic reproduction number (R_0_) in order to quantify the extent of disease spread. The R_0_ estimates were significantly different for the two spatio-temporal clusters of ASF in Italy, and they identified the two infected areas based on the time necessary for the number of cases to double (td) and on an exponential decay model. These results (R_0_ = 1.41 in Piedmont and 1.66 in Lazio) provide quantitative knowledge on the epidemiology of ASF in Italy. These parameters could represent a fundamental tool for modeling country-specific ASF transmission and for monitoring both the spread and sampling effort needed to detect the disease early.

## 1. Introduction

African swine fever (ASF) is a hemorrhagic disease caused by a DNA virus (ASFV) of the *Asfaviridae* family, causing high mortality in domestic pigs and wild boar or feral swine, with devastating ecological and socio-economic implications [1,2]. ASF is classified as a notifiable disease, and it is listed in the terrestrial Animal Health Code [3] and European Animal Health Law [4].

The main transmission routes of ASF are contact between infected and susceptible animals and through contaminated carcasses [5,6,7]. As demonstrated by several works, the carcasses of infected animals, ASFV-contaminated habitats, and tools or other mechanical vectors are the main risk factors that, if correctly managed, allow for the spread of the disease to be controlled or limited [6,8]. Furthermore, the long-term persistence of ASF at a low wild boar density is strictly related to direct and carcass-mediated infection [6,9]. Furthermore, the early detection of ASF during the initial spread of the virus in a susceptible population (i.e., the invasion phase) could determine the evolution of the epidemic [10]. Considering the absence of a licensed vaccine or a specific treatment against ASFV, the target actions recommended by the EU legislation on domestic pig populations comprise the depopulation of affected farms; the contact tracing of animals and animal products; and the establishment of protection and surveillance zones around the affected area’s premise, along with disinfection, movement restriction, and active surveillance [11]. The recommendations for wild populations include the definition of infected and surveillance zones, active carcass search and removal, the installation of fences, and intensive wild boar depopulation [12]. More precisely, a lack of carcass search and removal could favor the generation of an ASF endemic context [13,14,15].

In European countries, the first spread of ASFV genotype I (1960–1995) from Spain and Portugal to other countries in Western Europe resulted in its eradication, except for in Sardinia, where the virus has remained endemic for more than 40 years [16,17,18,19]. From 2007, ASFV genotype II was introduced through Georgia and spread to neighboring countries (Armenia, Azerbaijan, Russia, and Belarus) [20,21]. In 2014, the first cases in wild boars were reported in Lithuania, and several cases were subsequently reported in Estonia, Latvia, and Poland and, more recently, in the Czech Republic (2017), Romania (2017), Hungary (2018), Bulgaria (2018), Belgium (2018), Serbia, the Russian Federation, and Slovakia (2019) [22,23]. In 2020, Greece and Germany reported their first case in a domestic pig and a wild boar, respectively [24,25].

As previously described [26], in 2021 (December), the first ASF case was detected in the mainland of Italy (outside Sardinia) by passive surveillance activities. As mentioned above, Sardinia was the only region in Italy historically affected by genotype I, and it was subject to level IV restrictions until 2021. Currently, the Sardinian epidemiological situation has improved, with evidence of the absence of viral circulation, thanks to the implementation of a specific ASF eradication program [27]. During the first six months of the Italian ASF epidemic, a total of 220 genotype II ASF outbreaks were reported in two main areas defined as infected [28]. From 2020, a specific ASFV surveillance program, mainly based on passive surveillance, was introduced on a regular basis in Italy [26]. National data arising from this program are collected and archived on the specific Italian veterinary informative system for food safety (SINVSA).

Furthermore, after the first reported case, several actions aimed at eradicating the disease were taken inside the infected area, including the prohibition of hunting and other outdoor activities, active searching and disposal of wild boar carcasses, the depopulation of at-risk farms, and the stoppage of animal movements [26,29]. Otherwise, the incursion of ASFV in the mainland of Italy would threaten not only the areas included in the infected or surveillance zones but the entire country, given the need to enforce surveillance with the aim of contingent early detection in order to avoid new incursions or the further spread of the virus. This aim becomes even more difficult considering the fundamental role of human actions in the long-distance transmission of the disease, as reported in several countries [30,31,32,33]. As reported in Belgium and the Czech Republic, monitoring and surveillance actions, even in non-affected areas, are essential to eradicate the disease from the wild boar population [31,34,35,36,37].

While the importance of describing the epidemic curve in the early phase of disease incursion is a well-established concept [25], most of the time, the available data in the initial phase of disease spread are only related to dead animals, making it impossible to apply a Susceptible–Exposed–Infectious–Recovered (SEIR) model. Furthermore, during the incursion phase, it is of paramount importance to estimate the number of expected dead pigs or wild boar in order to set and evaluate the required sampling effort. The expected number of carcasses to be detected in a specific time–space is a function of the R_0_ parameter, which is defined as the average number of secondary cases caused by one infectious individual during its entire infectious period in a fully susceptible population [38]. As previously demonstrated, the progress of an epidemic increases in terms of speed and space rate, thus increasing the value of R_0_ [39]. In addition, monitoring how R_0_ changes over time can help identify shifts in transmissibility due to environmental or evolutionary changes and, hence, to elucidate the ecological and evolutionary drivers of disease emergence [40,41].

The aim of this work was to estimate ASF’s doubling time and the R_0_ from the data obtained during the first seven months of the Italian epidemic (from 29 December 2021 to 30 July 2022) in both the infected areas. The estimated parameters allow for the quantification and comparison of the spread of the infectious disease, the prediction of its speed, and the evaluation of the passive surveillance effort currently in place [38].

## 2. Materials and Methods

### 2.1. Data Source

The first ASF case in Italy was detected in a dead wild boar found by passive surveillance activities in the Ovada municipality, the Alessandria province in the Piedmont region, on 29 December 2021 [26]. As disposed by the EC regulation 2021/605 and based on the last EC provision 2022/1413, two main areas were defined as infected and subject to level II and III restrictions, with the associated surveillance zones subject to level I restrictions.

Considering the presence of ASF in wild boar populations, the northern infected area (PL, 2,884 km^2^) is subject to level II restrictions. This area includes 84 municipalities in the Piedmont region and 36 municipalities in the Liguria region, as well as 95 neighboring municipalities subject to level I restrictions. The southern area (LA, 515 km^2^) was established by the EC provision 2022/717 after the detection of the first ASF-positive wild boar on 4 May 2022 and was revised by the EC provision 2022/920 after the first detection of ASFV in a domestic pig farm (9 June 2022). This area is subject to level III restrictions, typical of areas where ASF involved both wild boar and domestic pigs, and it includes the area of the capital (Rome) within the administrative boundaries of the local health authority (ASL RM1). A total of 19 municipalities neighboring this infected area constitute the surveillance zone subject to level I restrictions. These two infected zones (PL and LA) were used as study areas.

The data included in this study were collected from the main official sources: the Italian Veterinarian National Database (BDN), the Veterinary Information Systems of the Italian Ministry of Health (VETINFO), and SINVSA. The information recorded was related to all suids tested for ASFV: the location (region, province, municipality, latitude, and longitude); the date of sampling; the date of notification if the presence of ASFV was confirmed by PCR+; the species (i.e., wild boar or domestic pig); the age and sex of the tested animal; the type of sample (i.e., spleen, blood, tonsil, kidney, or lymph node); and whether the sample arose from breeding, carcasses, or wild boar killed by road traffic. The state of the carcass was established during sample collection, based on the stage of conservation, and it was recorded as fresh, in decomposition, or in advanced decomposition, according to the FAO manual on ASF in wild boar [12]. Antibody detection was not carried out in any of the two areas. For the purpose of this study of representing the pattern of the disease over time, only reports related to PCR+ fresh carcasses were considered [42]. Furthermore, to avoid the bias related to the difference in the spread of ASF in wild species and domestic pig populations bred on farms, only wild boar carcasses (i.e., animals found dead) were considered, excluding culled animals, domestically bred pigs, and wild boars killed by road traffic accidents [13,15].

### 2.2. Estimation of Epidemiological Parameters

Combined techniques previously tested by studies with similar aims [42,43,44,45,46] were applied to estimate the basic reproduction number (R_0_) of the first ASF epidemic in Italy.

The R_0_ was estimated from the doubling time [44], assuming that the number of secondary cases increases exponentially, and the ASFV infectious period (*T*) of 5–7 days was assumed [46].

Visual data inspections and the Kolmogorov–Smirnov test were used to assess whether the data fitted the pre-specified distribution (i.e., exponential). If the data were not exponentially distributed, the subset data that best fit the pre-specified distribution were selected by a specific algorithm. The algorithm first log-transformed different subsets of these data and then modeled the subset using a linear model [42]. Finally, the model that maximized the Adjusted R-squared (Adj-R^2^) and minimized the prediction error in terms of the lowest residual standard error (RSE) and the lowest Bayesian Information Criterion (BIC) was chosen as the subset that better fit the linear distribution. Subsequently, two exponential (decay) models with a trend of distribution stabilizing over time [47] of the equation

(1) $$y=\alpha *e^{\beta x+\theta }$$

were fitted using the subset data from the PL and LA areas.

The estimated model parameters (*α*, *β,* and *θ*) were used to predict the expected number of carcasses (*y*) on the day (*x_t_*) in which we expected to find twice the number of carcasses on day *x*_1_. The difference between the two values resulted in the doubling time (*td*), which was related to R_0_ (new infections per generation) and the infectious period (*T*) by the equation:

(2) $$R_{0}=1+\left(\frac{T}{td}\right)\times log_{e}2$$

A parametric bootstrap was applied to estimate the confidence intervals for *td*. Then, we repeated the process of R_0_ estimation over 1000 resampling iterations to obtain a large set of likely R_0_ values. We displayed these R_0_ values in a histogram format, and we computed the mean and 95% confidence interval (95% CI) ranges for the R_0_ values.

All statistical analyses were performed using R software (Version 4.1.2, R-Foundation for Statistical Computing, Vienna, Austria).

## 3. Results

**Figure 1:**
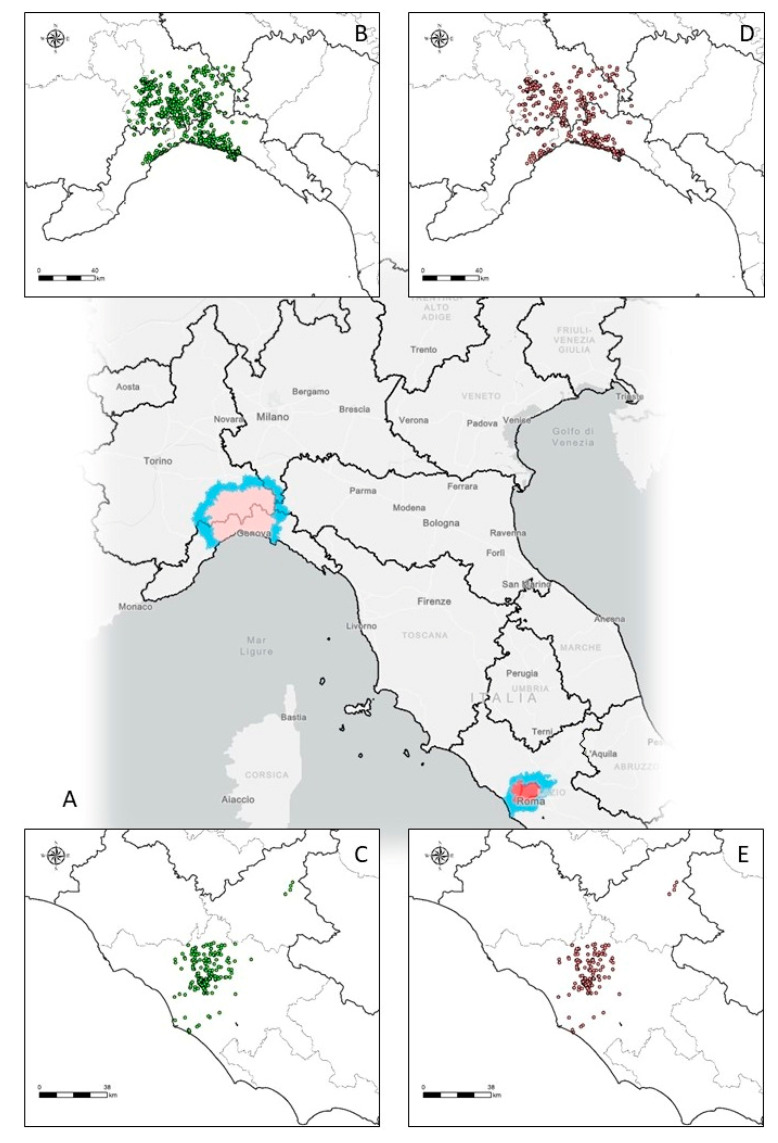
African swine fever epidemiological situation in Italy in the two infected and surveillance areas subject to level I (blue area), II (pink area), and III (red area) restrictions (**A**); wild boar samples from Piedmont (PL) area (**B**) and from Lazio (LA) area (**C**) and those from fresh carcasses in PL (**D**) and in LA (**E**). Data and associated information were collected by SIMAN.

From 29 December 2021 to 30 July 2022, a total of 6,632 wild boars and domestic pigs were sampled from the whole Italian territory, of which 1338 were from the two infected areas (Figure 1A). Most of the samples (1306, 98%) were from wild boars (1019 in PL and 287 in LA), as reported in Figure 1B,C.

A total of 220 ASF outbreaks (219 in wild boar and 1 in a domestic pig farm) were reported from 29 December 2021 to 31 July 2022 in the study area (173 in PL and 46 in LA), and they were grouped by the day of sampling, with an overall observation period of 214 days. As reported in Table 1, 51% (n = 467) of the samples collected in PL and 58% (n = 136) of those collected in LA were females.

Through a dentition evaluation, as described by Matsche in 1967 [48], most of the sampled animals in the PL area were determined to be adults (n = 379, 37%), while in the LA area, they were determined to be young (n = 148, 52%). In PL, 40% (n = 411) of the samples tested for ASF were from active surveillance, collected by hunting activities (n = 217) or road traffic accidents (n = 194), while 59% (n = 574) of the samples tested were from passive surveillance. Most of the carcasses included as passive surveillance were collected by volunteers or forest corps (n = 574), while only 34 were collected during the active search of carcasses on fields by regional organized patrols. In LA, 66% (n = 191) of the tested animals were from active surveillance (133 killed by road traffic and 58 hunted), and 33% (n = 96) were from passive surveillance. Only one carcass was found during the active search of carcasses on fields, while 95 were voluntarily reported by locals.

The same proportion of PCR+ animals (17% and 16%) was found in PL and LA, with almost all of them (170 in PL and 45 in LA) from passive surveillance, confirming the fundamental role of this activity. In PL and LA, 40% (n = 68) and 38% (n = 17) of the animals found dead were adults, respectively.

In PL, 416 (90%) carcasses had a fresh conservation status, while in LA, 50% (n = 48) were at this stage of conservation, but 42% (n = 40) were in decomposition. All the rest were mummified.

Overall, 91 samples referred to as PCR+ fresh carcasses (72 in PL and 19 in LA) were included in the final analysis.

### Doubling Time and R_0_ Estimation in PL and LA Infected Areas

**Figure 2:**
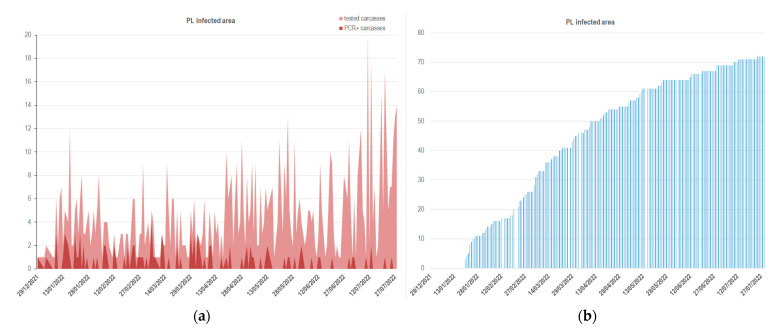
The time series illustrate (**a**) the number of samples from each type of carcass with ASF collected (light red curve) and the number of samples from fresh carcasses tested as PCR+ in Piedmont-Liguria, and (**b**) the cumulated frequencies of these samples over time.

**Figure 3:**
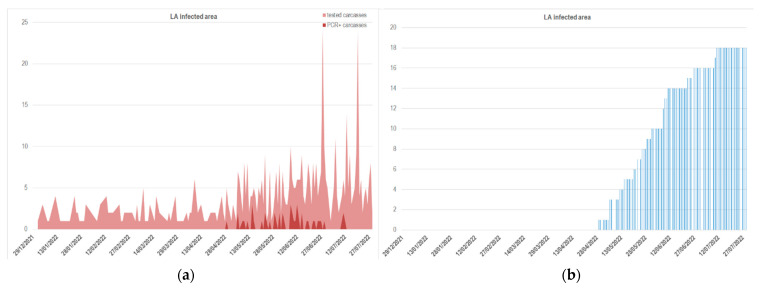
The time series illustrate (**a**) the number of samples from each type of carcass with ASF collected (light red curve) and the number of samples from fresh carcasses tested as PCR+ in Lazio, and (**b**) the cumulated frequencies of these samples over time.

After the first wild boar carcasses were detected as being positive for ASF by PCR, an active search for carcasses was implemented in both the infected areas. The active search for carcasses was mainly aimed at understanding where the virus came from, starting at the edge of the infected areas. Most of the carcasses in the inner part of the infected areas were found by volunteers. A mean of 0.6 (SD = 0.8) ASF-positive carcasses per day in the PL area (Figure 2a,b) and a mean of 0.5 (SD = 0.4) ASF-positive fresh carcasses per day in LA (Figure 3a,b) were detected over a mean of 4.7 (SD = 3.4) and 2 (SD = 3.4) carcasses (fresh, in decomposition, and mummified) PCR-tested in one day in PL and LA, respectively.

**Figure 4:**
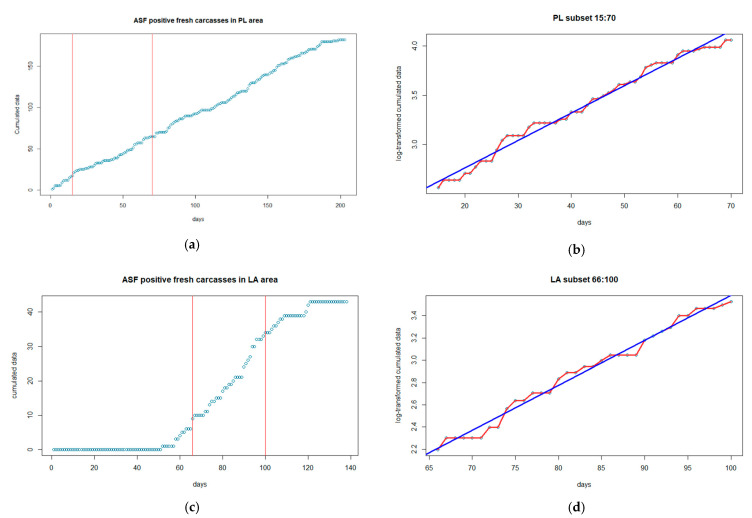
Subset selection: (**a**) subset selected for PL data, including observational times 15:70 (from 26 January to 22 March 2022), based on cumulated data of ASF-positive fresh carcasses; (**b**) linear model fitting based on the log-transformed cumulated data from the PL subset 15:70; (**c**) selected subset for LA data, including observational times 66:100 (from 15 May to 19 June 2022), based on cumulated data of ASF-positive fresh carcasses; (**d**) linear model fitting based on the log-transformed cumulated data from the LA subset 66:100.

Considering that the complete data were not exponentially distributed and that the Kolmogorov–Smirnov test was statistically significant (*p*-value ≥ 0.05) in both the PL and LA datasets, the subsets of data that best fit an exponential distribution were detected. Following the example of Belgium and the Czech Republic proposed by Marcon et al. in 2019 [42], the cumulated data from PL and LA were log-transformed, and different subsets of these data were modeled using linear models to choose the subset that better fit the linear distribution (i.e., highest Adj-R^2^, lowest RSE, and lowest BIC). The subset selected as the most appropriate to fit the model for the PL area included data from day 15 (26 January 2022) to 70 (22 March 2022) (Figure 4a,b). This subset of data exhibited a good fit with the linear model, as demonstrated by the highest Adj-R^2^ value of 0.987 (RSE = 0.052). Considering the delay in the incursion of ASF in Lazio four months later, the selected subset that best fitted the linear model included data from the 66 (15 May 2022) to the 100 (19 June 2022) log-transformed observations (Figure 4c). As shown in Figure 4d, these subsets fitted a linear model well, with the highest Adj-R^2^ value equal to 0.986 (RSE = 0.049).

**Figure 5:**
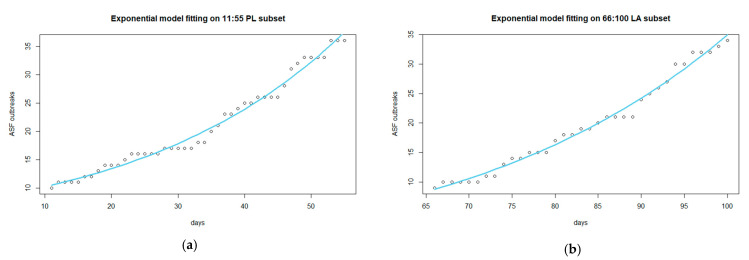
Model fitting for the selected subset 11:55 for Piedmont-Liguria infected area (**a**) and for 66:100 subset selected for Lazio infected area (**b**).

The cumulative distributions of the ASF outbreaks reported during the selected period in PL (Figure 5a) and LA (Figure 5b) are closest to an exponential distribution, the parameters of which are reported in Table 2.

**Figure 6:**
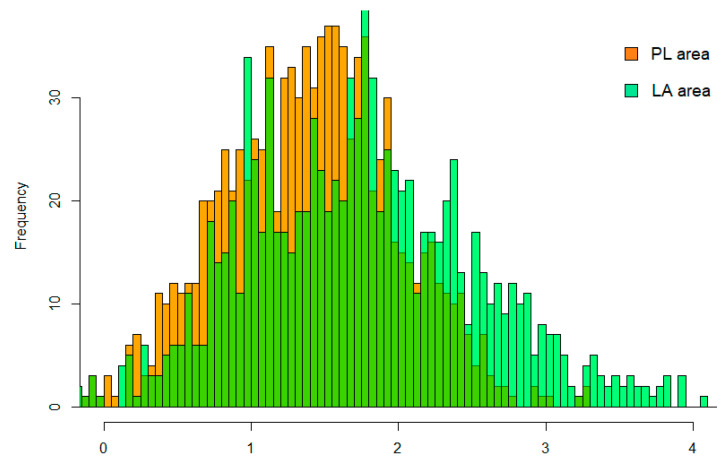
Histogram distribution of the PL R_0_ values (orange) and LA R_0_ values (green).

Computing the *td* as described above, its estimated value was equal to 11.89 days in PL and 8.33 in the LA area. Thus, solving R_0_ using Equation (2), the estimated mean value of this parameter was 1.41 [95% CI = 1.37–1.45] for the PL infected area. In the LA infected area, the low *td* necessary to observe a higher number of cases produced a mean R_0_ value equal to 1.66 [1.61–1.75], indicating a faster disease spread. The distribution of these R_0_ values are displayed as a histogram in Figure 6.

## 4. Discussion

After almost 40 years since the last detection of ASF in mainland Italy (excluding Sardinia), referred to as an isolated and quickly resolved case in 1983, the ASFV genotype II epidemic started from the same area in Piedmont. Even though the estimated wild boar density in this area is very high (i.e., >500 animals per 100 km^2^ [49]), the introduction of ASF infection is certainly attributable to unaware human action. In fact, this area is characterized by intensive commercial trade, given the proximity to the Genoa port [25]. Otherwise, the high wild boar density and the ongoing hunting season during the detection of the first ASFV case could be considered determinant factors for the disease spread.

The applied exponential model indicates a slightly higher R_0_ value in the LA area than in the PL area. Both of these values (1.66 and 1.41, respectively) are similar to those estimated on the Sardinian data from the first three years of the epidemic in Anglona (mean value = 1.14), they are in line with those estimated for wild boar populations in other countries [15], and they are similar to those estimated for herd transmission (mean value = 1.7) and for indirect transmission (mean value = 1.5) [50].

R_0_ is often used to quantify the spread of a disease and as an indicator of the potential magnitude of an epidemic. Its value is dependent on several variables related to both the disease and the host population. For diseases with a high case–lethality ratio, mortality cases can be used as a proxy for the number of newly infected individuals [51]. As demonstrated by several works, the speed of ASFV could be well-described by the main epidemiological parameters (i.e., R_0_, the force of infection, the doubling time, and the transmission rate) to compare the different spreading rates of ASF [50]. Mechanistic models have been successfully applied to ASF to design and evaluate targeted and alternative control strategies, and to elucidate epidemiological parameters. These methods are of great importance for the assessment of control strategies [50]. Furthermore, the estimation of epidemiological parameters could be a fundamental tool when attempting to plan an active search for carcasses, defining numerical and temporal goals, particularly in the first phases of disease containment [15,42,50].

However, estimation from real data is strictly affected by sampling effort and management. In Italy, the active search for carcasses was mainly aimed at the edge of the infected area, with the first aim being to understand as precisely as possible where the virus was located and with the secondary being to remove infected carcasses. Most of the carcasses in the inner part of the infected areas were found by volunteers and not through an active and systematic search, both in the LA and PL areas. All these factors, in addition to the ecological conditions and the wild boar population structure, could have affected the R_0_ estimation. Thus, the estimated value is the minimum R_0_, while it is very likely that the true R_0_ is higher.

In addition to the estimation of the epidemiological parameters, an assessment of the robustness of these estimations should be provided [42,50]. Remembering that, during the first years of an ASF epidemic, the key to fighting the spread of the disease is passive surveillance rather than hunting [13], additional effort should be dedicated to finding and removing as many wild boar carcasses as possible. Furthermore, this effort should be numerically evaluated and targeted based on the areas mainly at risk to avoid massive unnecessary expenses, as demonstrated by Desvaux et al. in 2021 [52]. The main goal could be to find and remove at least 80% of the carcasses during the first stage of the epidemic [13].

Although the estimated R_0_ values for the PL and LA areas are the minimum possible real values, it can be shown that ASF does not spread very fast, particularly in heterogeneous habitats. Indeed, the center of Rome (Lazio) is characterized by small parks separated by highways and highly built-up areas. Equally, PL has an orography and a distribution of railways, motorways, and national roads that still fragment the territory, therefore reducing the number of secondary cases and, consequently, the spread speed.

The spread of infectious disease is strictly related to the density of the susceptible population, which affects the number of instances of direct contact and, thus, disease transmission. However, it has been demonstrated that landscape fragmentation (i.e., the conversion and development of sites into urban areas, and the linkage of these sites via roads and railroads) is a major cause of the rapid decline of many wildlife populations [53,54,55]. This process generates isolated habitats that affect ecological interactions among animals, reducing their movement across the landscape, as well as potentially affecting metapopulation dynamics [56,57,58].

Furthermore, a few works on classical swine fever (CSF) hypothesized that spatial continuity is more important in disease spread than animal density. Thus, landscape fragmentation may reduce disease transmission [59]. This was well-described in 2020 by Dellicour et al. [31], and it was recently confirmed by Salazar et al. [60], highlighting the strong impact of the fragmentation of territories on ASF propagation. Environment and habitat fragmentation, as well as a robust estimation of the animal density, are factors that need a deep evaluation in Italy and in countries that have reported a recent ASF incursion.

## Figures and Tables

**Table 1:** Characteristics of the wild boar samples collected in the two main Italian infected areas from 29 December 2021 to 30 July 2022.

Features of the Samples	Piedmont-Liguria (n = 1019)	Lazio (n = 287)
Female	467 (51%)	136 (58%)
Age		
Young (0–6 months)	272 (27%)	148 (52%)
Subadult (6–18 months)	277 (27%)	56 (20%)
Adult (>18 months)	379 (37%)	81 (28%)
NA	91 (17%)	2 (9%)
Source of the sample		
Active surveillance ^†^	411 (40%)	191 (66%)
Passive surveillance *	608 (59%)	96 (33%)
PCR+ samples from active surveillance ^†^		
Young (0–6 months)	1 (33%)	0 (0%)
Subadult (6–18 months)	1 (33%)	0 (0%)
Adult (>18 months)	1 (33%)	0 (0%)
PCR+ samples from passive surveillance *		
Young (0–6 months)	22 (13%)	16 (35%)
Subadult (6–18 months)	38 (22%)	12 (27%)
Adult (>18 months)	68 (40%)	17 (38%)
NA	42 (25%)	0 (0%)
Carcass conservation stage		
Fresh	416 (90%)	48 (50%)
In decomposition	146 (24%)	40 (42%)
Advanced decomposition	25 (4%)	6 (6%)
NA	21 (3%)	2 (2%)
PCR+ fresh carcasses	72 (17%)	19 (20%)

^†^ refers to culled or hunted animals or those killed by road traffic accidents; * following the EFSA definition, passive surveillance only refers to animals found dead and not those culled by road traffic accidents [15].

**Table 2:** Estimated exponential model parameters.

Infected Area	α	β	θ	Doubling Time	R_0_ (95% Bootstrap Confidence Interval)
Piedmont-Liguria	26.14	0.016	−20.90	11.89 days	1.41 [1.37–1.45]
Lazio	1.74	0.031	−5.07	7.33 days	1.66 [1.61–1.75]

## Data Availability

All the data are reported in the main text.
